# The perceptual effects of signal components: black sword margins are crucial for signal size discrimination in green swordtails *Xiphophorus hellerii*

**DOI:** 10.1098/rspb.2024.2137

**Published:** 2025-01-29

**Authors:** Eleanor M. Caves, Laura A. Kelley

**Affiliations:** ^1^ Department of Ecology, Evolution, and Organismal Biology, Brown University, Providence, RI 02912, USA; ^2^ Department of Ecology, Evolution, and Marine Biology, University of California, Santa Barbara, Santa Barbara, CA 93106, USA; ^3^ Centre for Ecology and Conservation, University of Exeter, Penryn, UK

**Keywords:** perceptual processing, signal evolution, visual ecology, mate choice, swordtail, melanic coloration

## Abstract

The signals that mediate mate choice can be complex, comprising multiple components, and understanding how complex signals evolve under sexual selection has been the focus of much study. However, open questions still remain about the role of the female’s sensory and perceptual processes in shaping the evolution of complex signals. Male green swordtails *Xiphophorus hellerii* have an elongated caudal fin that comprises colour, length and a black melanic margin; females prefer males with larger bodies, longer swords and complete black sword margins. Here, we used a two-choice assay to quantify female preferences for animations of courting males of different sizes with or without sword margin coloration, and found that, when a black melanic margin was present, females exhibited preferences for larger males. However, when the margin was absent, females did not show size-based mate preference, though females spent equal time assessing males in both treatments. Our results suggest that the presence/absence of the black sword margin is an important predictor of female preference, specifically a female’s ability to discriminate between potential mates of different sizes, pointing to a novel size discrimination function of black margins in animal signals, which in many species involve patterns or structures with dark edges.

## 1. Introduction

A major focus in the study of sexual selection has been on the signals that females use to assess potential mates. Assessment signals can be complex, comprising multiple components such as pattern, colour, motion and sound, and much research has attempted to reveal why signals have certain components. Since the early 1990s, two main hypotheses have emerged that posited that assessment signal form might evolve due to selective pressures imposed by the signal receiver’s sensory system. The ‘receiver bias’ hypothesis [1,2] stated that signals should evolve forms that exploit the innate properties, or biases, of the receiver’s sensory system and brain, to increase the stimulation of a sensory system by a signal. Building on these ideas, the ‘sensory drive’ hypothesis [3] posited that, in addition to signal traits evolving particular forms due to sensory biases, both signal form and sensory capability should additionally be under selection to optimize transmission and detection in the environment where signalling takes place. Following detection and transduction, however, a number of perceptual and cognitive processes occur that impact how a receiver ultimately responds to a signal [4,5], but how perceptual processes impact signal evolution is highly understudied.

One important, but underexplored, hypothesis is that signal components aid in perceptual or cognitive processing by the receiver in ways that help receivers to accurately assess a signal trait and make decisions. Many assessment signals vary in size (e.g. the length of a feather or fin, the area of a colour patch or the size of a body), and in many systems, it has been shown that females assess the size of a male trait and exhibit directional preferences for traits of different sizes (e.g. [6–9]), most often for larger traits (reviewed in [10]) that typically reflect some aspect of male quality. The ability of a female to discriminate between various senders’ traits may be adaptive for her ability to choose the best-quality mate; alternatively, female preferences for larger males may arise as a result of sensory biases for objects that provide greater retinal stimulation (e.g. [11]). In either case, males may be under selection to evolve traits that increase their apparent size, and so some signal components may evolve to aid in a receiver’s ability to accurately assess size or discriminate between signallers of different sizes.

Many animals incorporate black coloration into their signalling traits (e.g. in birds [12]; in mammals [13] and in aposematic or warning coloration [14] in various taxa). Black coloration often surrounds or abuts a colour patch, which can have multiple perceptual impacts. First, surrounding a colour patch with darker coloration increases contrast and thus the patch’s detectability [15], maximizing the conspicuousness of a colour pattern [3]. In guppies *Poecilia reticulata*, black contours appear along colour patches during courtship, perhaps enhancing luminance contrast and accentuating colour patterns [16,17], and the removal of black reduces a male’s attractiveness [18]. Second, dark edges stimulate edge detectors in vertebrate eyes [19–21], aiding in object detection, recognition and localization, a fact that has been exploited by animals seeking to camouflage via disruptive coloration [22]. Lastly, conspicuous and highly contrasting traits, like colour patches surrounded by black edges, can also serve to draw a receiver’s attention [23]. Because attention can only be paid to one focal object at a time [24], drawing a receiver’s attention can be an important function of signal components [25], and evidence from humans suggests that visual attention can alter the perceived size of stimuli [26]. Therefore, black margins and edges may be perceptually salient during size discrimination tasks, but despite the widespread distribution of dark edges around colour patches or signalling traits, this idea remains unexplored.

Male green swordtails (*Xiphophorus hellerii*) possess an elongated caudal fin called a ‘sword’ that comprises an upper and lower black stripe (the black ‘margin’), and coloration (green, yellow or orange) between the black stripes. Females prefer males whose swords have black coloration over those lacking black coloration [27], and whose swords have complete as opposed to partial black coloration [28]. Evidence suggests the sword is an energetically inexpensive way of increasing the overall apparent size of the individual, as females prefer males with larger body sizes, specifically larger lateral areas [11]. Additionally, females perceive size differences proportionally, meaning that when discriminating between two males of different sizes, they assess their proportional (relative) size difference, rather than their absolute size difference [29]. Here, we use a two-choice behavioural paradigm (following [29]) to assess how the presence or absence of the black sword margin impacts female preferences for males of different sizes and provide evidence that black margins aid in size discrimination of male signalling traits by females.

## 2. Methods

### (a) Animal care and ethics

Animals in this experiment were treated in accordance with the ethical guidelines of the University of Exeter (ethics approval eCORN002243). Fish handling and experiments were carried out by EM Caves (Home Office Personal License I56658687) and P Prentice (I2099DA1E), under Home Office Project License PF6E68517. Fish used in this experiment were sexually mature descendants of a wild-derived population collected in Belize in 2002. Although females were originally housed in the laboratory in mixed-sex groups, at least one month prior to the start of the first experiment, females were moved to be housed in single-sex groups of 4−7 individuals in 30 l tanks. Fish were fed a mixture of bloodworm, mysis shrimp and artemia each morning and flake food (ZM Flake, Fish Food and Equipment, Hampshire, UK) each evening. Water temperature remained between 22 and 24°C and tanks were lit from above with AquaBeam LED lights (Tropical Marine Centre, Herefordshire, UK) on a 12:12 light:dark cycle. All fish were tagged subcutaneously with an individually identifiable combination of coloured elastomer tags (Northwest Marine Technology Inc, Washington, USA).

### (b) Behavioural experiments

Following the protocol previously used in [29], a two-choice behavioural paradigm was used to assess a female’s preference when animations of two courting males that differed in size were shown, and which did or did not have black coloration along the top and bottom margins of the sword fin (hereafter, ‘black margin’).

#### (i) Stimulus design

Stimuli were created using digital photographs since two-dimensional animations are a widely used tool to examine mate preferences in fish [30–32], including in swordtails [33] and in our specific swordtail population [29]. Stimuli were derived from a photograph of a male swordtail in the experimental population whose body (44 mm) and sword length (32 mm) were close to the mean in the population (mean ± standard deviation body size: 42.5 ± 5.31 mm; sword length: 28.5 ± 9.97 mm).

**Figure 1.**
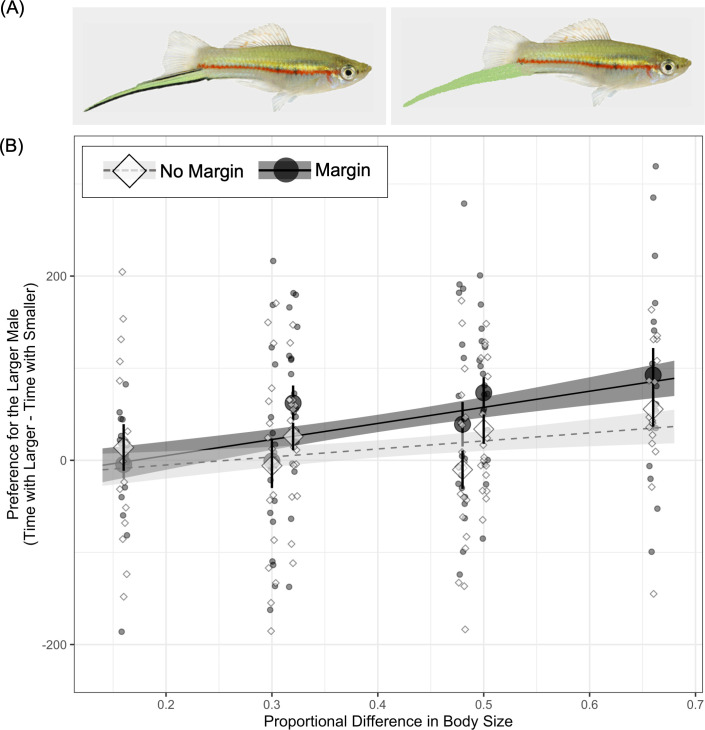
Females show a stronger preference for the larger male in a pair as their proportional size difference increases, although the trend is stronger when a black margin is present (A, left; B, black circles) versus absent (A right; B white diamonds). (A) Representative stimuli used in our experiment. In (B), large symbols show means, bars show standard error, smaller symbols show raw data and trendlines show fitted model estimates for a model of preference predicted by proportional difference for ‘margin’ and ‘no margin’ treatments (and with fish ID included as a random effect), with a 95% confidence interval (shaded areas). Note that all of the data for the ‘margin’ treatment were collected as part of a prior experiment [29].

After taking a digital photograph, the fish was separated from the background using Adobe Illustrator. The size of the fish was calibrated such that the size of the stimulus when displayed on the tablets used in trials (Samsung Galaxy Tab 10.1, Samsung Corp; 22.3 cm × 14 cm screen, 1200 × 1920 pixel resolution, 60 Hz refresh rate) equalled the size of the fish in real life. This original image was then scaled up or down to result in male stimuli of different lateral areas (hereafter, ‘body size’), following [34]. Identical versions of each stimulus, but without the black margin, were created by replacing the black margin on the sword with adjacent sword fin coloration using the rubber stamp function in Photoshop (figure 1*a*).

We animated the stimuli to represent courting males by importing them into a Microsoft PowerPoint (v. 16.57) slide with a blank, bright grey (RGB: 238, 238, 238) background (following [30]) and using PowerPoint ‘animation paths’. Animations were made to represent males swimming from one side of the tank to the other, moving off the screen, and reappearing swimming in the other direction over the course of 30 s (following [30,31,35]). We additionally animated the backward-swim manoeuvre, a courting behaviour performed by male *X. hellerii* [36], at a rate of three backward-swim manoeuvres every 60 s (per [36]) (electronic supplementary material). Stimulus and background colour, location of stimuli on the screen and path and speed of stimulus were identical across stimulus males, with only the size of the male and the presence/absence of the black margin varying.

Overall, we had six animated stimuli (table 1) with black margin coloration (the ‘margin’ treatment) and six identical animated stimuli without black margin coloration (the ‘no margin’ treatment). Stimuli were then presented in pairs (a|b, a|c, e|f, d|f, a|e, a|f; see electronic supplementary material, table S1 for details of each stimulus) within a treatment group—i.e. two ‘margin’ stimuli or two ‘no margin’ stimuli—in six combinations that met two criteria. First, all size differences were theoretically resolvable, given the visual acuity of female green swordtails (three cycles per degree [37]) and the size of the experimental tanks. Second, pairs covered a broad range of both proportional (range: 0.16–0.66) and absolute (169–691 mm^2^) differences in body size, with the absolute difference in body size calculated as


$$A_{l}-A_{s},$$

**Table 1.** Body length (given by standard length), sword length and lateral projection area of each stimulus used.

stimulus name	body length (mm)	sword length (mm)	lateral projection area (mm^2^)
**A**	53.6	38.9	1054
**B**	49.5	35.8	885
**C**	44	32	721
**D**	43.4	31.5	692
**E**	37.4	27.3	533
**F**	30.8	22.2	363

and proportional difference calculated as


$$\left(A_{l}-A_{s}\right)/A_{l},$$

where *A*_l_ is the area of the larger stimulus and *A*_s_ is the area of the smaller stimulus.

#### (ii) Two-choice trials

During two-choice trials, females (*n* = 24) were housed in groups of 4−7 individuals and were physically and visually isolated from males for at least one month prior to testing (following [11]). Immediately prior to a trial, a pair of females were moved together (due to ethical guidelines minimizing time spent in isolation) to a tank for 20 min in which they were in visual, but not physical, contact with males, to prime females for a mate choice task. Prior work has shown that females in our population, exposed to these conditions, exhibit behaviours indicating sexual receptivity to males [29].

Females were then placed individually in a two-choice tank (45.7 × 25.4 × 25.4 cm) filled to a depth of 15 cm using water from the home tank system and with a clump of Java moss in the centre as shelter. Trials were filmed from above using a Sunkwang C160 video camera with a 6−60 mm manual focus lens suspended above the tank. The camera was connected to a computer running the Viewer tracking software (BiObserve), which virtually divided the tank into three equally sized zones and tracked all movements made by the fish for the duration of a trial. To improve the accuracy of the automatic tracking, two-choice tanks were lit from underneath (following e.g. [38,39]) with a lightpad (UltraSlim LED LightPad, MiniSun, Manchester, UK). A cardboard screen was placed around the tank prior to the trial to prevent external visual disturbance.

Females were placed inside of a clear acrylic cylinder (15 cm diameter) in the centre zone of the tank for a 15 min acclimation period. During this acclimation, tablets were placed against each end of the tank, displaying only a plain grey background and a small amount of water from a tank housing males was added to the two-choice arena, to provide the female with olfactory cues from real males and further prime her for a mate choice task. Following the acclimation period, trials began with each tablet displaying 1 min of plain grey followed by 1 min of a male stimulus. During this period, the female was still constrained to the cylinder, to ensure that females viewed both male stimuli from the centre and thus from a consistent distance. The cylinder was then removed, allowing the female access to the entire tank while male stimuli continued to play at each end of the tank for three further minutes. Following trials, females were returned to their home tanks, and Viewer was used to extract the amount of time (in s) a female spent in each zone, i.e. spent with each male stimulus. After 48 h, the same procedure was followed with the same female and the same pair of male stimuli, but with each stimulus presented on the opposite side of the tank to account for possible side biases. All females were shown the stimulus pairs in the same order, and presentation order had no effect on female preference for the larger male in a pair, whether a male had a black margin or not, either in this study (electronic supplementary material, figure S1) or in a previous study with the same females [29].

All fish were first presented with the ‘margin’ stimulus as part of a different experiment [29]. Following that experiment females were kept together in groups; five months after completion of the first experiment, the entire experimental protocol was repeated as described above, except using male stimuli from which the black margin coloration had been removed. Given that all females had been tagged with individually distinctive combinations of coloured elastomer tags at the start of the previous study, we were still able to identify individuals during the second experiment.

In total, 24 females were presented with 12 stimulus pairs, with two trials per stimulus pair (over two 3-min periods). If a female did not leave the centre zone during a trial or exhibited stress symptoms (rapid darting back and forth across the tank) that trial was rerun at a later date, and if the female either did not leave the centre zone or exhibited stress again during the second run of a given trial, that female was excluded from further analysis for that comparison. Excluding some trials in which females were stressed resulted in data for between 16 and 23 females each of whom viewed all 12 comparisons (i.e. six pairs of males with the margin and six pairs of males without the margin) two times each, for a total of 544 trials for data analysis.

### (c) Statistical analyses

All analyses were run in R v. 4.0.3 [40]. We calculated preference for the larger male by taking association time (in s) with the larger male in a pair minus association time with the smaller male in a pair by summing times across both 3 min trials, to result in one association time value across the entire 6 min trial.

We then asked whether absolute or proportional difference—along with treatment—better described a female’s preference for the larger male by fitting linear mixed effects models using the *lmer* function of the package *lme4* [41] and using maximum likelihood. Because absolute and proportional differences are highly correlated (Pearson correlation coefficient, *r*_4_ = 0.90, *p* = 0.01), they likely should not be included in the same model [42]. Therefore, to avoid issues with collinearity, we could not include both absolute and proportional differences in the same model, and thus to compare which is a better predictor of preference and how that relates to treatment, we used a model comparison approach based on the Akaike information criterion corrected for small sample size (AICc), in which we built three model sets. In model set 1, the response variable was preference for the larger male in a pair and the predictor variable (fixed effect) was either proportional size difference, absolute size difference or treatment (margin/no margin). We then built additional models in which absolute or proportional size difference was combined with treatment, either in an additive fashion or as an interaction. Lastly, we built a null model with no fixed effects. Fish ID was included as a random effect in all models.

Models were ranked based on the AICc for small sample sizes [43,44], and we assigned ΔAICc values by calculating the difference between the AICc value of a given model and the AICc value of the best-fit model (the model with the lowest AICc value in that set). Following [45], ΔAICc values were used to calculate relative likelihoods for each model *i* within a set using the formula


$$l_{i}=exp\left[-\left(1/2\right)\Delta_{i}\right].$$

We then calculated model weight, the probability that each model *w_i_* within a set of models is the best, by dividing the likelihood of a given model *l_i_* by the sum of the likelihoods of all models within that set [45].

The above models suggested that treatment had a significant effect on female preference. To examine this further, we split the data into ‘margin’ and ‘no-margin’ datasets and for each dataset fit models (model sets 2 and 3) in which the predictor variable was either absolute size difference, proportional size difference or neither (the ‘null’ model). Fish ID was included as a random effect in all models, and as above, the model fit was assessed using AICc, and model weights were calculated.

The results of the above models suggested that female preferences for the larger male were much weaker in the ‘no margin’ treatment than the ‘margin’ treatment, a pattern which could either be explained by females simply not detecting (due to lower contrast), being interested in or associating with, males without a black sword margin. Therefore, we conducted two post hoc analyses to examine these possibilities. First, we built one further model in which the response variable was the total amount of time a female spent with any stimulus (the larger and smaller males combined) during both trials of each stimulus pair. Fixed effects were treatment and proportional difference, for which there are no collinearity issues and which thus can be included in the same model. Additionally, we did not have alternative models that we wished to compare, so here we were able to include both predictors in the same model and then assess the significance of the fixed effects. To assess the significance of each fixed effect by comparing the likelihood ratio of a full model, which included both fixed effects, to that of a model without each fixed effect in turn using the ‘drop1’ function in *lme4*. Second, we used paired *t*-tests (or paired two-sample Wilcoxon tests if paired differences were distributed non-normally) to statistically examine whether total time spent with both male stimuli combined differed significantly between the margin or no-margin treatment for a specific size comparison. Because of the paired nature of these tests, we could only use fish for which we had ‘complete’ data (i.e. trials for a given comparison in both the ‘margin’ and ‘no margin’ treatments).

## 3. Results

Females displayed stronger preferences for the larger male in a pair as the proportional difference in size between the two males increased, with the relationship between proportional size difference and preference appearing stronger for males in the ‘margin’ versus ‘no margin’ treatment (figure 1*b*; effect sizes given in electronic supplementary material, table S1). In support of this, the best-fit model in model set 1 had both proportional difference and treatment as fixed effects (table 2). The second best-fit model (ΔAICc = 0.94) additionally included the interaction between proportional difference and treatment (table 2).

**Table 2.** Summary of model response and predictor variables, ΔAICc values and model weights (*w_i_*). For each model set, the best-fit model is listed first. The asterisk indicates an interaction term. AICc: Akaike information criterion corrected for small sampple size.

model	ΔAICc	*w_i_*
**model set 1**		
preference ~ proportional difference + treatment + (1| fish ID)	0.00	0.41
preference ~ proportional difference * treatment + (1| fish ID)	0.94	0.26
preference ~ proportional difference + (1| fish ID)	2.52	0.11
preference ~ absolute difference + treatment + (1| fish ID)	2.87	0.10
preference ~ 1 + (1| fish ID) (null model)	4.45	0.04
preference ~ absolute difference * treatment + (1| fish ID)	4.69	0.04
preference ~ absolute difference + (1| fish ID)	5.31	0.03
preference ~ treatment + (1| fish ID)	7.77	0.01
**model set 2**		
margin data: preference ~ proportional difference + (1| fish ID)	0.00	0.83
margin data: preference ~ absolute difference + (1| fish ID)	3.60	0.14
margin data: preference ~ 1 + (1| fish ID) (null model)	6.14	0.03
**model set 3**		
no margin data: preference ~ absolute difference + (1| fish ID)	0.00	0.36
no margin data: preference ~ proportional difference + (1| fish ID)	0.10	0.34
no margin data: preference ~ 1 + (1| fish ID) (null model)	0.28	0.30

We then examined the ‘margin’ and ‘no margin’ data separately and found that for the margin data, the best-fit model included proportional difference as a predictor and had 83% of the model weight, followed by the ‘absolute difference’ model (ΔAICc = 3.6, weight = 0.34) and then the null model (ΔAICc = 6.14, weight = 0.04). By contrast, for the no margin data, all three models were within ΔAICc of 0.28 of one another and had roughly equal weight (table 2), implying that neither proportional nor absolute size difference explained the preference data any better than no predictors at all. Together, these results suggest that when a margin is present, females proportionally assess size differences and prefer larger males, but when the margin is absent, females do not exhibit a size-based preference.

**Figure 2.**
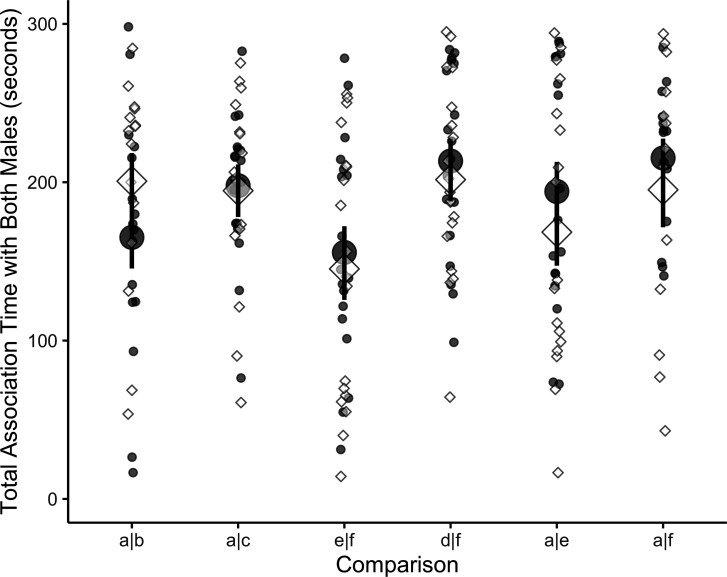
Females showed no significant differences in total time spent with males between the ‘margin’ (black circles) and ‘no margin’ (white diamonds) treatments, for a given comparison. Large symbols represent means, bars show standard error and small symbols show raw data. Stimulus pair names are ‘male 1|male 2,’ with ‘a’ being the largest male and ‘f’ being the smallest. Comparisons are arranged from smallest to largest proportional differences. See electronic supplementary material, table S1 for the size of each male in mm^2^. Sample size for each comparison a|b (*n* = 17), a|c (*n* = 19), e|f (*n* = 20), d|f (*n* = 20), a|e (*n* = 19) and a|f (*n* = 16).

Lastly, we examined whether size differences did not significantly predict preferences in the ‘no margin’ treatment because females did not approach or associate with males with no black margin. A model showed, however, that only proportional difference, and not treatment, was a significant predictor of total time spent with both stimuli combined (proportional difference: estimate ± standard error = 71.8 ± 29.1, *t* = 2.47, *p* = 0.01; treatment: estimate ± standard error = 1.33 ± 9.0, *t* = 0.15, *p* = 0.88). In support of this, total time spent with any male did not differ significantly between the ‘margin’ and ‘no margin’ treatment for any comparison (comparisons listed in the format stimulus 1|stimulus 2, *t*-tests or Wilcoxon tests; a|b: *t*_16_ = 1.54, *p* = 0.14; a|c: *t*_17_ = 0.92, *p* = 0.37; e|f: *V* = 92, *p* = 0.92; d|f: *t*_19_ = −0.89, *p* = 0.38; a|e: *t*_18_ = −0.94, *p* = 0.36; a|f: *t*_15_ = −0.52, *p* = 0.61; figure 2).

## 4. Discussion

Our results point to the presence/absence of the black sword margin being an important predictor of female preference, specifically a female’s ability to discriminate between potential mates of different sizes and display a preference for the larger male. In particular, when the margin is present, females prefer larger males in a pattern best described by proportional processing, but when the margin is absent, females do not appear to exhibit size-based mate preferences at all. The lack of observed preference for larger males when the black margin is absent does not appear to be driven by females not detecting, being uninterested in, or not recognizing, males without a margin as potential mates. We found that females spent equivalent amounts of time associating with male stimuli in both the ‘margin’ and ‘no margin’ treatments, indicating that females were sampling or assessing mates for equal amounts of time in both treatments, but then only exhibiting a preference for the larger male when a margin is present. To our knowledge, this is the first demonstration of a size discrimination function for melanic margins in an animal signal, with implications for signal evolution across a range of taxa.

Many signals—including many mating signals—are complex, or comprise multiple components. A large body of literature has demonstrated that the components of a complex signal may combine to impact preference, which has been demonstrated in multicomponent signals such as those in junglefowl *Gallus gallus* [8] and mice *Mus domesticus* [46], as well as multimodal signals (e.g. in *Schizocosa* wolf spiders [47] and songbirds [48]). In some cases, these preferences appear to arise because the different components of a multicomponent signal each allow a female to assess an aspect of a male’s condition. This occurs, for example, in male peacock trains, where females prefer both longer trains (which are correlated with fat reserves) and trains with more eyespots (which are correlated with muscle mass) (e.g. [49,50]). In other cases, however, evidence suggests that female preferences may be underlain by the sensory or perceptual effects that components have on one another. For example, one component can act as an amplifier that reinforces female assessment of a second, informative component [51]; it can act to direct or hold a female’s attention (reviewed in [25]); or it can aid in a female’s ability to assign a receiver to a category (e.g. [52]). Here, we provide support for the idea that female preferences for a signal component—the black margin—may be due to the function of those margins as a size-discrimination aid.

Previous work [29] has shown that female green swordtails discriminate between males of different body sizes based on proportional, rather than absolute, size differences between them, consistent with Weber’s Law (also known as ‘proportional processing’). Under proportional processing (reviewed in [53]), a perceptual system’s ability to discriminate between stimuli of different magnitudes is based upon the proportional difference between them rather than the absolute difference. As a result, a given absolute difference can more readily be discriminated if two stimuli are low-magnitude than if they are high-magnitude because the proportional difference between the two low-magnitude stimuli is greater.

In general, proportional relationships are highly salient to sensory systems [53]. Many perceptual processes are thought to have arisen in order to help sensory systems reduce and efficiently process the enormous variation in magnitude that can occur within one sensory parameter [54]. Compressing information into a logarithmic scale is one way to efficiently code magnitude differences, and it is this coding efficiency that appears to be the root of Weber’s law. We speculate here that the sword margin aids in size discrimination, which in this system is known to be proportional, although melanic margins might similarly aid in absolute discrimination as well. Precisely what the mechanism is by which black margins aid in size discrimination, however, is still an open question. Retinal edge detectors in vertebrate eyes are stimulated by dark edges, which helps with object detection, recognition and localization, all steps that are probably necessary in order to assess size and size differences [19–21]. Beyond the retina, contrasting colour patterns also function to draw a viewer’s attention [23], and visual attention has been shown to be important in size assessment [26]. Thus, how sword margins aid in size discrimination, while an open question, is likely due to both retinal-level and higher order processes.

Functionally in a signalling context, for example during mate choice, proportional processing means that receivers can more readily discriminate between low-magnitude signallers than between high-magnitude signallers, which is consequential given that low-magnitude signallers are often low-quality and less preferred than high-magnitude signallers (reviewed in [10]). One predicted effect of proportional processing on signal evolution is that it may favour signal elaboration or innovation [53,55]. Under proportional processing, when comparing males that are high magnitude, females cannot easily discriminate or notice an even further increase in magnitude. Thus, if the cost of producing large signals is high, then the cost-to-benefit ratio of producing an even higher magnitude signal may quickly become unfavourable for a male, since female perceptual processing may mean it does not result in any additional female preference. As a result, new traits or signal components may be favoured to evolve in order to support female assessment of the signal. In humans, attention has an important influence on an object’s perceived size [26], and attention across taxa has been shown to be guided by high-contrast markings such as dark margins [23]. Thus, one hypothesis is that the black margin has been ‘added’ to the sword signal, which additionally comprises both size and colour, to draw female attention and aid in size discrimination, an important process for females seeking to choose the highest quality mate. Recent molecular evidence [56], however, suggests that the black margin may be the ancestral state in the genus *Xiphophorus*, and thus that, rather than selection favouring its gain in certain species, it may have been lost in some. Comparative studies across the family Poeciliidae, in which black signal components, including margins, are highly variable, but preferences for them are widespread (e.g. in guppies [18,57] and southern platyfish *X. maculatus* [58]), could help to clarify the mechanisms and selective pressures underlying our results.

One important caveat to emphasize regarding our findings is that, due to a number of scientific, logistical and animal ethics considerations, we were unable to run this experiment in a fully factorial design, and thus there is no way to entirely disentangle order effects or shifts in preference due to ageing or experience that may have occurred between collecting the ‘margin’ and ‘no margin’ data, which occurred five months apart. However, within each treatment, experiments took between three and four months to complete, and during that time, we did not see any shifts in preference (electronic supplementary material, figure S1), so we think that the effect of time between treatments was likely minimal. Additionally, fish of a range of ages were used in each treatment, so although all fish did age between the ‘margin’ and ‘no margin’ experiments, we did not only test young fish on stimuli with a margin and old fish without. Lastly, prior to each experiment, females experienced similar housing in single-sex tanks and then visual stimulation by males immediately before each trial, and females were not housed with males between the ‘margin’ and ‘no margin’ experiments, meaning we have no reason to believe that females were exposed to males in a way that would alter their preferences prior to one experiment over the other. However, we suggest that future experiments should use fully factorial designs to test perceptual effects, both to confirm these findings and to examine whether perceptual processing varies with age.

Overall, though studies have previously demonstrated preferences in green swordtails for males with a black sword margin (e.g. [27,28]), ours is the first to show that the ability to discriminate between males of different sizes depends upon the presence of the black margin. Thus, we have here provided evidence for a novel function of black margins: size discrimination. Complex, multicomponent signals are widespread across taxa, but the hypothesis that components have evolved as a result of their perceptual effects on female receivers is understudied. Given that many signals serve to convey a signaller’s size, and that dark signal margins are relatively common, we suggest that components enhancing size discrimination ability in receivers may be widespread, not only in mating signalling but also in other contexts like aggressive and territorial signalling.

## Data Availability

All data and codes have been uploaded and are publicly available on the Dryad Data Repository [59]. Supplementary material is available online [60].
